# The Application of eHealth in Symptom Management of Patients With Breast Cancer During Endocrine Therapy: Scoping Literature Review

**DOI:** 10.2196/92030

**Published:** 2026-06-04

**Authors:** Chengjia Gu, Lili Shen, Shiyu Zhang, Nan Zhang

**Affiliations:** 1 Comprehensive Breast Health Centre, Ruijin Hospital, Shanghai Jiao Tong University School of Medicine, 197 Rui Jin Er Road, Shanghai, 200025, China, 86 602268 ext 13661819697; 2 Department of Nursing, Ruijin Hospital, Shanghai Jiao Tong University School of Medicine, Shanghai, China

**Keywords:** eHealth, endocrine therapy, symptom management, Preferred Reporting Items for Systematic Reviews and Meta-Analyses, PRISMA, patient with breast cancer

## Abstract

**Background:**

Endocrine therapy is a fundamental treatment for hormone receptor–positive breast cancer. However, treatment-related symptoms may lead to poor adherence and premature discontinuation, adversely affecting clinical outcomes. eHealth technology interventions have been widely applied as potential tools to support patient education, symptom management, and adherence.

**Objective:**

This review aimed to evaluate the application, effectiveness, and limitations of eHealth technologies in symptom management among patients with breast cancer undergoing endocrine therapy.

**Methods:**

A systematic literature search was conducted across 9 databases, including MEDLINE Complete, Academic Search Complete, PubMed, and CINAHL Ultimate, covering studies published between February 2015 and February 2025. Keywords included “breast cancer,” “endocrine therapy,” and “ehealth,” along with synonyms and related terms. Eligible studies examined the use of eHealth interventions in patients with breast cancer undergoing endocrine therapy. From 1352 records, 30 (2.2%) studies were included after screening and full-text review.

**Results:**

The 30 included studies comprised 13 (43.3%) randomized controlled trials, 7 (23.3%) single-arm studies, 3 (10%) retrospective analyses, 2 (6.7%) observational studies, 3 (10%) mixed methods studies, and 2 (6.7%) qualitative studies. eHealth interventions, primarily mobile apps, telemonitoring systems, and SMS text messaging, demonstrated benefits in short-term symptom management, such as reductions in hot flashes, joint pain, anxiety, and sexual distress and improved medication adherence. However, evidence regarding long-term adherence and quality of life outcomes remains inconsistent. Considerable heterogeneity existed in study designs, adherence measures, and intervention components. Nurses were considered key contributors in implementing and monitoring eHealth interventions.

**Conclusions:**

eHealth interventions show promise in improving symptom management and short-term adherence among patients with breast cancer receiving endocrine therapy. Methodological limitations and variability across studies restrict the strength of conclusions. Future research should prioritize long-term outcomes, standardized measures, and integration into routine clinical practice while incorporating perspectives from multiple stakeholders.

## Introduction

Breast cancer is heterogeneous, with approximately 80% of cases classified as hormone receptor positive [1,2]. Adjuvant endocrine therapy (AET), primarily selective estrogenic receptor modulators and aromatase inhibitors, is a standard treatment for patients that significantly reduces the risk of recurrence and mortality [3-5]. Clinical guidelines recommend 5 to 10 years of AET [6-8]. Despite its clinical benefits, adherence to endocrine therapy remains suboptimal. Studies indicate that up to one-third of patients discontinue treatment within 5 years, often due to treatment-related adverse effects, inadequate information, comorbidities, or patient preferences [9,10]. Common adverse effects include hot flashes, joint pain, osteoporosis, sexual dysfunction, weight gain, sleep disturbances, depression, anxiety, and cognitive impairment [6,11]. These symptoms can impair quality of life (QOL) and contribute to nonadherence, ultimately affecting prognosis [12]. Therefore, effective symptom management is critical for improving adherence and clinical outcomes.

The World Health Organization has introduced the Global Strategy on Digital Health, emphasizing the integration of information and communications technologies to improve health outcomes [13]. In this review, eHealth refers to the use of digital tools such as mobile apps, SMS text messaging, telemedicine platforms, web-based portals, wearable devices, and electronic patient-reported outcome systems for real-time symptom monitoring, medication reminders, patient education, automated alerts, and remote self-management support [14]. Over the last 2 decades, eHealth has been increasingly used to support symptom monitoring, patient education, and remote care delivery [15]. Previous research has demonstrated the value of eHealth in chronic disease and cancer management, including symptom tracking, adherence support, and self-management [16,17].

Patients with breast cancer have used eHealth technologies for issues such as pain, sleep disorders, lymphedema, psychological distress, and chemotherapy side effects [18-22]. However, no comprehensive scoping review has synthesized evidence specifically on eHealth applications for symptom management during endocrine therapy. This review addresses that gap by evaluating the current evidence on effectiveness, strengths, limitations, and implementation considerations to inform clinical practice.

## Methods

### Search Strategy

This review followed the PRISMA (Preferred Reporting Items for Systematic Reviews and Meta-Analyses) guidelines (Checklist 1) to ensure thorough search quality and standardized processes [23,24]. Nine databases were searched: MEDLINE Complete, Academic Search Complete, CINAHL Ultimate, Health Source: Nursing/Academic Edition, APA PsycInfo, PubMed, Scopus, Embase, and Web of Science. The search used combinations of terms for “breast cancer,” “endocrine therapy,” and “eHealth” supplemented with synonyms, academic terms, abbreviations, and related terminology. Boolean operators (“AND” and “OR”) were applied to combine terms for search strategy construction systematically. Limits included English-language, peer-reviewed articles focusing on the application of eHealth in breast cancer and published between February 2015 and February 2025. The full search strategy is detailed in Table 1.

**Table 1.** Summary of the search strategy for the literature review.

Domain	Description
Search keywords	
Breast cancer	“Breast Neoplasms” OR “Breast Tumor,” OR “Breast Cancer” OR “Mammary Cancer” OR “Malignant Neoplasm of Breast” OR “Breast Malignant Neoplasm” OR “Malignant Tumor of Breast” OR “Breast Malignant Tumor” OR “Cancer of Breast OR Mammary Carcinoma, Human” OR “Human Mammary Neoplasms”
Endocrine therapy	“endocrine therapy” OR “endocrine treatment” OR “aet” OR “hormone therapy”
eHealth	ehealth OR e-health OR telecare OR telemedicine OR telehealth OR “digital health” OR mHealth OR teletherapy OR “smartphone App” OR “smartphone application” OR “health apps” OR “remote monitoring” OR “mobile app” OR “mobile application” OR “wearable devices” OR telemedicine OR tele-care OR telecare OR “Medicine, Virtua” OR “Tele Referral” OR tele-referrals OR tele-referral OR “Virtual Medicine” OR “Health, Mobile” OR “Mobile Health” OR telehealth OR “Electronic App” OR “Electronic Application” OR “Application, Mobile” OR “mobile phone” OR “smartphone” OR “text-, electronic-“ OR “multimedia-message” OR “electronic mail” OR “phone applications” OR “computer” OR “podcast” OR “videos” OR “internet” OR “website” OR “chatroom” OR “message board” OR “activity tracker” OR “electronic health” OR “mobile health” OR “telemedicine”
Other search parameters	
Search limits	2015-2025
Type of material searched	English-language peer-reviewed scholarly articles
Databases searched	EBSCOhost databases (Academic Search Complete, Health Source: Nursing/Academic Edition, CINAHL Ultimate, MEDLINE Complete, and APA PsycInfo), PubMed, Scopus, Web of Science, and Embase

### Eligibility Criteria

Studies were eligible for inclusion if they were empirical research articles that examined the application of eHealth interventions specifically for symptom management or related outcomes, including medication adherence and QOL, among patients with breast cancer undergoing endocrine therapy in either the early or advanced disease stages. Exclusion criteria included animal studies, research protocols, review articles, conference abstracts, non–English-language publications, and studies that focused exclusively on other cancer treatments rather than endocrine therapy.

### Study Selection and Data Extraction

A total of 1352 records were retrieved from the 9 databases, and 148 (10.9%) were removed based on the predefined inclusion and exclusion criteria. The remaining 1204 records were imported into the Zotero software (version 7; Corporation for Digital Scholarship), where 616 (51.2%) duplicates were removed. Among the remaining 588 records, title and abstract screening resulted in the exclusion of 504 (85.7%) studies, primarily due to irrelevant populations (non–breast cancer or non–endocrine therapy; n=295, 58.5%), irrelevant focus (chemotherapy only, screening, or survivorship care without endocrine therapy; n=110, 21.8%), nonempirical content (reviews, protocols, and abstracts; n=59, 11.7%), or focus on other diseases (n=40, 7.9%). Subsequently, of the remaining 84 records, 30 (35.7%) were excluded because the full texts could not be retrieved. Full-text assessment was then performed on the remaining 54 articles, of which 24 (44.4%) were excluded because they described only study protocols or were conducted on survivors of breast cancer without a specific focus on endocrine therapy. Ultimately, 30 articles met all eligibility criteria and were included in this review (see Table S1 in Multimedia Appendix 1 [25-54] for details). Data extracted included study design, participant characteristics, intervention details, outcomes (symptom management, adherence, QOL, and patient experiences), and key findings.

### Risk-of-Bias Assessment

Two oncology nurse researchers assessed the risk of bias in randomized controlled trials (RCTs) using the Cochrane risk-of-bias tool independently [55]. Discrepancies, if any, were resolved by a third reviewer. Due to heterogeneity in designs, interventions, and outcome measures, no meta-analysis was performed. The Critical Appraisal Skills Programme checklists were used to evaluate methodological quality across study types [56].

### Data Synthesis

Findings were synthesized thematically into three main aspects: (1) effectiveness and limitations of eHealth for symptom management and adherence, (2) the role of nurses, and (3) patient experiences and impact on QOL.

## Results

### Overview

**Figure 1.**
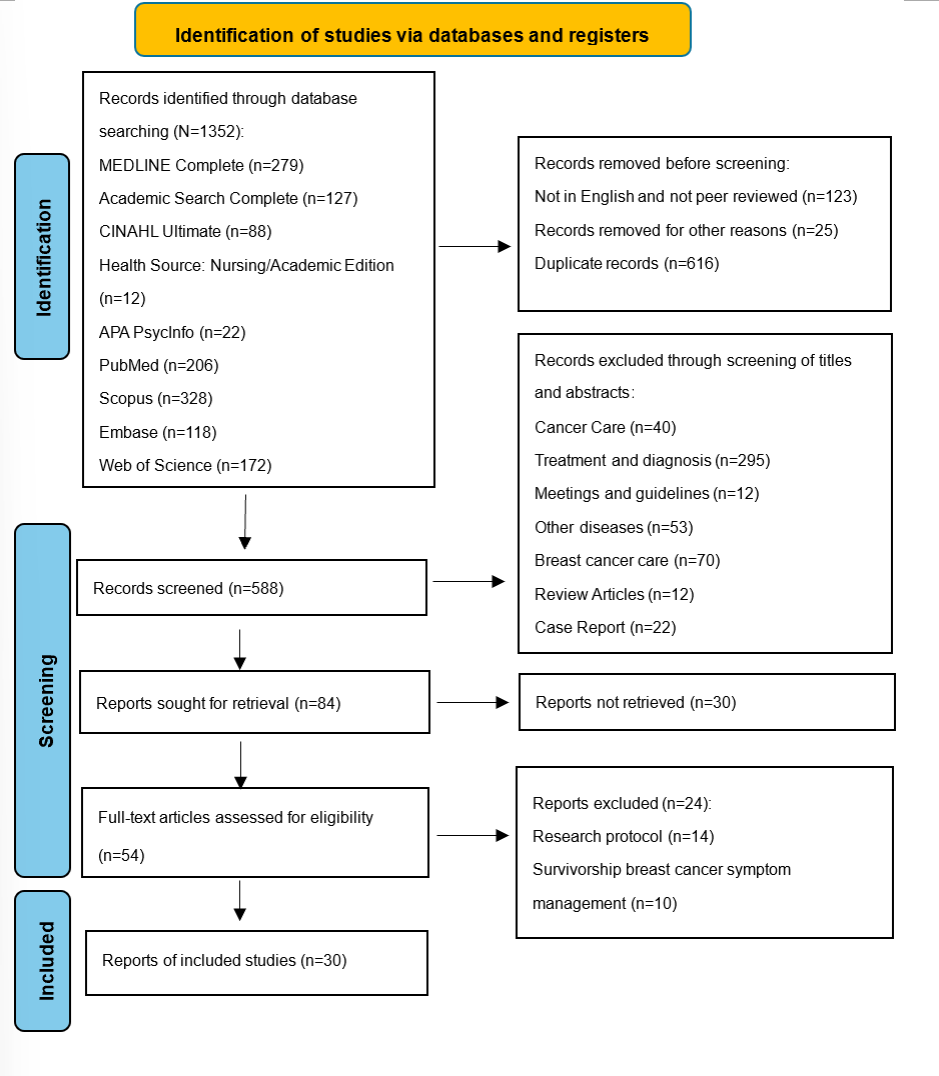
PRISMA (Preferred Reporting Items for Systematic Reviews and Meta-Analyses) 2020 Fflow Ddiagram of the literature review.

The 30 selected studies included 13 (43.3%) RCTs, 7 (23.3%) single-arm studies, 3 (10%) retrospective analyses, 2 (6.7%) observational studies, 3 (10%) mixed methods studies, and 2 (6.7%) qualitative studies. Most studies (n=17, 56.7%) were conducted in the United States, followed by Sweden (n=3, 10%); Japan and the United Kingdom (n=2, 6.7% each); and Germany, Turkey, Singapore, South Korea, and China (n=1, 3.3% each). Three key themes emerged from the review: (1) effectiveness and limitations regarding symptom management and adherence to endocrine therapy, (2) nurses’ role in eHealth interventions, and (3) experiences of patients with breast cancer with eHealth tools and the impact on QOL. The PRISMA flow diagram is presented in Figure 1.

### Effectiveness and Limitations of eHealth in Symptom Management

eHealth interventions, including mobile apps, telemonitoring, and SMS text messaging, facilitated timely self-reporting of symptoms and triggered alerts for health care providers. In total, 10% (3/10) of the studies allowed patients to self-report and record symptoms using mobile apps [29-31], whereas another 13.3% (4/30) of the studies used questionnaires to record patients’ symptoms [32-35], allowing for real-time data capture and proactive management.

A total of 20% (6/30) of the studies reported positive effects on symptom control using eHealth [36-41]. One RCT combining an educational website with telephone counseling significantly reduced sexual difficulties, distress, and anxiety compared with usual care [36]. Another RCT using a mobile education program with nurse reminders improved physical well-being, pain, emotional functioning, and endocrine-related symptoms at 3 months [57]. Teleconferencing interventions in 2 RCTs enhanced self-efficacy and reduced hot flashes and depression [41,42]. A longitudinal study of remote tai chi sessions via an app demonstrated reductions in joint pain, anxiety, and hot flashes after 12 weeks [38]. An app-based intervention in advanced endocrine treatment improved arm symptoms, cognitive function, anxiety, and depression [39]. The mobile app integrated daily exercise logging, medication adherence tracking, Fitbit activity data synchronization, personalized goal setting, educational modules on hormonal therapy side effects, and weekly progress visualizations, with automated feedback and nurse coach messaging. These eHealth tools enabled more accurate and timely symptom tracking, supporting personalized care plans and reducing symptom burden in the short term (typically 1 to 6 months) [29,31,35,42]. However, most studies focused on short-term outcomes and specific subgroups, such as patients with poor AET adherence [41], recent sexual activity [36], or aromatase inhibitor–induced arthralgia [38], limiting the generalizability of the findings. Patients with breast cancer on endocrine therapy often experience complex, interacting symptom clusters [22]; few interventions (1/30, 3.3%) addressed this holistically.

### Medication Adherence

Medication adherence is commonly defined as taking medication as prescribed; nonadherence includes delays, dose deviations, or premature discontinuation [58]. Previous studies have shown that eHealth interventions have a significant impact on medication adherence in patients with chronic diseases and those taking medication for mental health conditions [59-61]. Medication adherence is addressed solely in relation to its direct correlation with effective symptom management; premature discontinuation is a subsequent result of inadequately controlled symptoms. Therefore, studies showing improved adherence were interpreted strictly as evidence of effective symptom control rather than as a separate end point.

Retrospective studies indicated 5-year adherence rates of 48% to 53%, primarily due to adverse events and symptoms [43-45]. Access to practical information, active monitoring of side effects, and effective medication reminders have been suggested to improve adherence [62]. A total of 16.7% (5/30) of the studies found that digital health tools, including SMS text messages, mobile apps, and emails, that incorporated symptom education, self-monitoring, and alerts, improved medication adherence [31,32,34,46,47]. Another 16.7% (5/30) of the studies reported benefits from reminder-based educational interventions [30,39,48,49]. However, other studies reported inconsistent results. In total, 15.4% (2/13) of the RCTs with 1- and 3-year follow-ups on SMS text messaging found no significant differences in adherence [33,50]. One retrospective study with telephone follow-up showed no long-term benefit [45], and one RCT on a mobile program reported no improvement despite educational modules [40]. Medication adherence was measured across several aspects: prescription fill rates of more than 80% [32,39], self-reports [31], estrogen level monitoring, and discontinuation rates (Table 2). The heterogeneity in measures and data collection and analysis methods contributed to variability in the findings. Improvements were mainly evident in short-term studies; long-term data remain limited, highlighting the need for sustained interventions.

**Table 2.** Summary of medication adherence measures in the literature review.

	Mougalian et al [32]	Hershman et al [33]	Tan et al [50]	Park et al [48]	Takada et al [31]	Smith et al [34]	Wheeler et al [49]	Chan et al [39]	Krukowski et al [47]	Jacobs et al [51]
Prescription fill rate exceeding 80%	✓							✓		
Integrating urine analysis with self-reporting		✓								
Combining self-reporting with innovative bottle technology							✓			
Using estrogenic level measurements in conjunction with a simplified medication adherence report			✓							
Patient self-records					✓					
Discontinuation rates						✓				
Integrating prescription fill data with smart bottle opening data				✓					✓	
Medication adherence reporting scale+smart pill bottle										✓

### Nurses’ Role in eHealth for Symptom Management

Nurses were the primary implementers in most interventions, leveraging eHealth to enable real-time symptom assessment, 2-way communication, prioritization of high-risk cases, and referrals [63-66]. A total of 13.3% (4/30) of the studies found that nurses enabled 2-way communication and referral by prioritizing high-risk symptoms [32,34,40,47]. Nurses sharing information through eHealth tools could improve both patient satisfaction and the quality of care [52,53]. Nurses delivered education via SMS text messages, app modules, and teleconferences, enhancing patients’ self-management skills and symptom control [29,33,37,41,67]. Moreover, the included interventions used dynamic, standardized, and structured assessments of both cognitive and somatic symptoms, which new data indicate are superior to conventional static questionnaires for earlier detection of clinically relevant changes and timely intervention [67].

Nurse involvement improved care efficiency, multidisciplinary collaboration, and resource use [68-70]. In total, 10% (3/30) of the studies, which were nurse led, showed that nurse-led approaches improved high-quality health care and reduced high-cost contacts and outpatient visits [46,47,53]. However, challenges included increased workload, role ambiguity, and the need for process adaptation [69,71]. Most evidence reflects patient perspectives; fewer studies (2/30, 6.7%) examined nurses’ or physicians’ acceptance and barriers. Cost-effectiveness evaluations were largely absent, underscoring the need for health economic analyses.

### Patients’ Experiences With eHealth Tools and the Impact on QOL

In this review, patients generally expressed high satisfaction and acceptance of SMS text message reminders [32,50], teleconferencing [37], and mobile apps [29,54], citing ease of use, helpful information, and improved self-management (satisfaction often of >90%). Combined tools such as videoconferencing applications and wearables further enhanced satisfaction, and many patients were willing to continue using them [38,47,62]. A total of 20% (6/30) of the studies examined QOL impacts. Four of these studies reported significant improvements in various domains such as physical, emotional, or endocrine-specific typically after 1 to 6 months of using eHealth tools such as mobile apps with reminders or tele-education [31,39,51,52]. One study providing tele-education showed significant improvements in patients’ QOL after a 3-month intervention [52]. Another study used a teleconference education format and demonstrated significant improvement in patients’ QOL after 24 weeks of intervention [51]. However, 2 RCTs found no significant differences in QOL: one study implemented a mobile app with SMS text message reminders and follow-up assessments at 6 and 12 months [40], whereas the other integrated the app with a patient e-reporting intervention and assessed QOL at 3 months after the intervention [35].

Heterogeneity and variations in QOL study results likely stem from differences in QOL instruments [31,35,37,39,40] (Table 3). Various QOL measurement scales may yield different results when evaluating due to differences in study design, cultural appropriateness, and assessment dimensions [72-74]. The assessment of QOL in survivors of breast cancer requires a multidimensional approach encompassing physical, psychological, social, and spiritual well-being, indicating that the use of a single assessment tool may not fully capture the heterogeneity in their QOL trajectories [75]. This review found that eHealth interventions effectively improved patients’ QOL in the short term. However, the long-term effects and trends for patients with breast cancer undergoing endocrine treatment remain to be thoroughly researched and explored.

**Table 3.** Summary of questionnaires assessing quality of life (QOL) in the literature review.

Study	FACT-ES QLS^a^	EORTC QLQ-C30^b^	EORTC QLQ-BR23^c^	FACT-B^d^	12-Item Short Form Health Survey	Validity and reliability	Measurement results
Çınar et al [52], 2021	✓					✓	There was a statistically significant difference in the total FACT-ES QLS scores (*P*<.05).
Jacobs et al [51], 2022				✓		✓	There was a significant improvement in QOL in the intervention group at 24 wk (*P*<.01).
Takada et al [31], 2022				✓		✓	The intervention did not negatively affect QOL.
Chan et al [39], 2024		✓	✓			✓	Subscale scores were significant: *P*=.02 for cognitive functioning, *P*=.04 for future prospective functioning, and *P*=.04 for arm symptom score.
Graetz et al [40], 2024					✓		Not statistically significant.
Okuyama et al [35], 2024				✓		✓	Not statistically significant.

a FACT-ES QLS: Functional Assessment of Cancer Therapy–Endocrine Subscale Quality of Life Scale.

b EORTC QLQ-C30: European Organisation for Research and Treatment of Cancer Quality of Life Questionnaire Core 30.

c EORTC QLQ-BR23: European Organisation for Research and Treatment of Cancer Quality of Life Questionnaire–Breast Cancer Module.

d FACT-B: Functional Assessment of Cancer Therapy–Breast.

However, several limitations remain in sample selection when examining patients’ experience with eHealth interventions. In terms of sample inclusion, the mixed methods study by Jacobs et al [37] of patients with high levels of anxiety involved predominantly White patients with high symptom burden. Additionally, the single-arm study by Gomaa et al [38] only included patients who reported joint pain. Some studies (4/30, 13.3%) predominantly selected patients who were younger, White individuals, more educated, and economically privileged [32,38,40,47]. In terms of sample size, 2 mixed methods studies and 1 single-arm study lacked a control group. They had small sample sizes of 15 [29], 10 [54], and 39 [47]. Furthermore, several studies with low response rates (3/30, 10%) were noted [46,52,53]. The main reasons for this problem included lack of personalized interventions, poor user interface design, time constraints, technical difficulties, and privacy issues [32,47,49]. Therefore, the characteristics of nonresponding patients and patients who declined to be enrolled may introduce selection bias.

## Discussion

### Principal Findings

This is the first scoping review to synthesize evidence on eHealth applications specifically for symptom management in patients with breast cancer undergoing endocrine therapy, incorporating effectiveness, adherence, nurse roles, patient experience, and QOL. Interventions varied (mobile apps: 8/30, 26.7%; SMS text messaging: 5/30, 16.7%; telemedicine: 6/30, 20%; online portals: 3/30, 10%; hybrid electronic patient-reported outcomes+wearables: 2/30, 6.7%; remote tai chi: 1/30, 3.3%) but shared fundamental features: real-time data capture, threshold-triggered alerts, and remote feedback. Short-term benefits for symptom burden were consistent across app- and telemedicine-based approaches, although direct comparisons were limited. This comprehensive perspective not only facilitates an in-depth understanding of the mechanisms and actual effects of eHealth interventions but also provides valuable reference information for future clinical practice and research.

This review demonstrated eHealth’s efficacy in symptom monitoring and short-term management, with some positive effects on psychological symptoms and potential indirect benefits for adherence. However, many studies of symptom monitoring and management (4/30, 13.3%) applied single-arm designs with short follow-up periods, and adherence outcomes relied heavily on self-report, introducing bias [30-32,39]. The RCTs included in this review often lacked blinding, and interventions frequently targeted single symptoms or specific subgroups, overlooking complex symptom clusters and socioeconomic factors [33,40,47,48,50]. Nevertheless, this review found that eHealth interventions could improve patients’ medication adherence and short-term symptom management. However, current research relies heavily on one-sided quantitative data from patients while neglecting to assess the early warning systems of physicians and nurses.

This review revealed that nurses played a pivotal role in implementation, education, and coordination, contributing to efficiency and patient engagement. However, challenges such as workload, role clarity, and limited exploration of health care providers’ perspectives persist. Patient experiences were largely positive with high satisfaction, but generalizability is constrained by limited studies and equipment requirement issues [76,77]. QOL improvements were evident in the short term but inconsistent in the long term, partly due to measurement heterogeneity. In addition, most existing studies have examined the roles and functions of physicians and nurses in eHealth interventions primarily from the patient’s perspective. However, as the primary implementers of these technologies, health care providers’ own acceptance of eHealth tools and the barriers they encounter in daily practice remain underexplored.

This review indicates that eHealth interventions are generally associated with favorable patient experiences, high uptake rates, and positive short-term effects on QOL among patients with breast cancer receiving endocrine therapy. Prior literature similarly underscores this population’s expressed need for digital support. Nevertheless, several limitations constrain the generalizability of these findings. Many quantitative studies (4/30, 16.6%) featured small, relatively homogeneous samples, often restricted to participants with the necessary devices and digital access [31,38,39,49]. Low response rates to eHealth applications in some studies (6/30, 20%) further restricted applicability across diverse populations. These issues can hinder timely symptom detection and intervention for certain patient groups. Moreover, few studies have thoroughly explored patients’ specific needs in terms of tool design, particularly with respect to cultural adaptability.

The strengths of this review lie in its comprehensive literature search and the inclusion of diverse study designs, including RCTs, single-arm studies, retrospective analyses, observational studies, mixed methods studies, and qualitative research. Quality appraisal was conducted using the Critical Appraisal Skills Programme checklists, and the complementary nature of these methodologies, where RCTs help establish causality and qualitative studies provide rich insights into patient experiences, enhances the overall credibility of the synthesis. However, notable limitations were evident in the primary studies. Most (23/30, 76.6%) lacked adequate sample diversity and representativeness. In addition, the evidence base was heavily skewed toward high-income countries, with minimal data from low- and middle-income settings or rural areas, thereby limiting global applicability and scalability. Although the use of theories, models, and frameworks has been shown to strengthen the scientific rigor and explanatory depth of digital health research [78], only 13.3% (4/30) of the studies in this review were explicitly guided by such frameworks. Furthermore, most of the included RCTs (10/13, 76.9%) lacked blinding of participants or researchers, and 26.7% (8/30) of the studies used single-arm designs without control groups, both of which increase the risk of bias and reduce the robustness of the findings. In addition, the search period ended in February 2025, which may have omitted more recent high-impact publications. The restriction to English-language peer-reviewed articles may also have excluded relevant studies published in other languages. Despite these constraints, eHealth interventions demonstrate considerable potential to reduce symptom burden and improve adherence during endocrine therapy. Future research should prioritize long-term efficacy, greater equity in access, multi-stakeholder perspectives, and integration into routine oncology workflows to maximize real-world impact and sustainability.

### Conclusions

eHealth interventions offer promising support for symptom management and short-term adherence among patients with breast cancer receiving endocrine therapy, with generally high patient acceptance. Nurses are central to successful implementation. However, evidence is limited by methodological heterogeneity, short follow-ups, inconsistent long-term outcomes, and a lack of diversity. By addressing individualized patient needs and incorporating experiences from multiple stakeholders (patients, physicians, and nurses), eHealth tools can be optimized to reduce symptom burden, improve adherence, and enhance clinical outcomes. Future studies should prioritize rigorous designs, standardized measures, long-term follow-up, and equitable implementation to realize the full potential of digital health in oncology care.

## Supplementary material

10.2196/92030 Multimedia Appendix 1 Summary of main characteristics of identified research, including research aim, outcome measures, and results.

10.2196/92030 Checklist 1 PRISMA-ScR Checklist.

## Abbreviations

AET: adjuvant endocrine therapy
PRISMA: Preferred Reporting Items for Systematic Reviews and Meta-Analyses
QOL: quality of life
RCT: randomized controlled trial
