# Supplementary material for: The Application of eHealth in Symptom Management of Patients With Breast Cancer During Endocrine Therapy: Scoping Literature Review
Source: JMIR Cancer. 2026 Jun 4;12:e92030. doi: 10.2196/92030 (PMC13235842; doi:10.2196/92030)
Supplement: Multimedia Appendix 1 [file cancer-v12-e92030-s001.docx]

| **Table S1.** Summary of main characteristics of identified research, including research aim, outcome measures and results | | | | | | |
| --- | --- | --- | --- | --- | --- | --- |
| Author/Year | Research Aim | Methodology and Data Collection | Sample | Major findings | Recommendations | Strengths and Limitations |
| Krok- Schoenet al., /2019 | This study tested the feasibility and efficacy of using a text-based intervention to increase initiation,decreasediscontinuation,  and improve adherence as prescribed to adjuvant hormone therapy (AHT) among  hyphenatepost-menopausal breast cancer survivors. | **Methodology: Single-arm before-and-after controlled**  **study (pilot)**  The study used a 3-month intervention consisting of daily text messages with medication reminders and weekly interactive surveys via a smartphone app.  **Data collection**:  Baseline surveys: Before AHT started, participants completed baseline surveys  over the phone that assessed physical symptoms, cancer recurrence concerns,  self-efficacy, depression, pain disturbances, fatigue disturbances, and more.  2. Weekly Surveys: Participants completed medication adherence surveys once a week via the app.  3. Blood samples | The study recruited39 eligible postmenopausal breast cancer patients. They were asked to own a smartphone and agree to receive a text message intervention for three months. Ultimately, 27 patients (69.2%)  completed all study requirements  (including a baseline survey,  an exit survey, two blood collections, and no more than two weekly surveys missed). | 1. Medication adherence: Self-reported medication adherence significantly increased (P = 0.015).  2. Mental health function: As measured by the MCS-8, mental function showed significant improvement  (P = 0.007).  3. Perceived pressure: A significant reduction in perceived pressure was observed (P = 0.04).  4. Hormone levels:  Oestradiol, estrogenic, and estrone levels significantly decreased from baseline to study completion (P < 0.001), suggesting accurate patient self-reporting of AHT adherence.  5. Patient and physician feedback: 91.9% of patientsand 100% of physicians deemed the intervention beneficial. | The findings suggest that app-based interventions can increase AHT adherence and improve patient mental health. Future studies should conduct  larger randomized controlled trials  to validate this  intervention's effectiveness further and explore its impact on AHT adherence and quality of life. | **Limitation:**  1. Absence of a control group complicates assessing the intervention's isolated impact on AHT adherence.  2. Limited sample size.  3. Smartphone dependency, potentially  excluding eligible patients.  4. Recruitment timeframe constraints, precluding participation for patients already on AHT.  **Strengths:**  1. Multicentre design.  2. Integration of objective and subjective data.  3. High patient acceptance.  4. Cost-effectiveness and ease of implementation. |
| Jacobs et al./ 2022 | This study aimedto evaluate the feasibility,  acceptability and preliminary effects of a telemedicine- based intervention  (STRIDE) on symptom management, psychological distress and medication adherence during adjuvant endocrine therapy (AET) in breast cancer patients. | **Methodology:** randomized controlled trial  The intervention consisted of six weekly group videoconferences and two individual telephone follow-  up visits. The control group had routine care and medication monitoring only.  **Data Collection:**  1. Baseline data: Patients' clinical information was collected through the electronic health record  (EHR).  2. Post-intervention data3. Objective medication adherence data: patients' medication intake was recorded by MEMS Caps. | One hundred patients reporting AET-related distress were randomly assigned to either the STRIDE intervention group or the medication-monitoring (MedMon) control group.  Patients were also required to report AET-related distress (as assessed by the NCCN distress thermometer). | 1. Feasibility and acceptability: Patient satisfaction with the intervention was high, with95% of patients saying their needs were met.  2. Symptom distress: Compared with the control group, patients in the STRIDE group reported significantly less trouble with hot red symptoms at 12 weeks (p=.048), and  substantially less trouble with overall symptoms at  24 weeks (p =.007).  3. Quality of life: Patients in the STRIDE group experienced significant improvement in quality of life within 24 weeks (p <.001).  5. Anxiety symptoms: In the STRIDE group, anxiety symptoms were significantly reduced within24 weeks | The findings suggest that the STRIDE intervention has  the potential to  improve AET-related symptom distress, quality of life,  and coping skills in breast cancer patients. | **Strengths:**  1. High feasibility and  acceptability  2. Multidimensional evaluation  3. The intervention is based on various theoretical models (e.g., medication adherence frameworks, cognitive models of coping with cancer, etc.)  **Limitations:**  1. Insufficient sample diversity  2. Neither researchers nor participants were blinded to the groups,  which could introduce bias.  3. Measurement of medication adherence  4. Some patients could  not attend group meetings due to scheduling problems. |
| Gomaaet al./ 2022 | The purpose of  this study was toevaluate the safety, feasibility, acceptability and potential effects of remote TaiChiintervention (TaiChi4Joint)  in relieving AI-induced arthralgia in breast cancer patients | **Methodology：** one-arm longitudinal pilot study  Participants attended tai chi  classes thrice a week for 12 weeks via the Zoom platform.  **Data Collection：**  1. Baseline data  Post-intervention data: At 1, 2, and 3 months, participants completed the following questionnaire: WOMAC (Western Ontario and McMaster University Osteoarthritis Index). AUSCAN (Australian Canadian Osteoarthritis Hand Index). BPI (Brief Pain Inventory), FSI (Fatigue Symptom Inventory),HFRDIS  (Hot Flash Related Daily Interference Scale),PSQI  (Pittsburgh Sleep Quality Index), CES-D (Centre for Epidemiological Studies-Depression). | The study recruited39 eligible breasts who were female, ≥18 years of age, diagnosed with stage 0-III breast  cancer, treated with AI for at least 2 months and reported a joint pain score of ≥4 on a 0-10 scale. | 1. Feasibility and acceptability: 22 out of 39 patients completed the  3-month follow-up, with a retention rate of 56 percent  2. Symptom improvement: From baseline to 3 months,participants showed significant improvement in multiple symptoms: WOMAC pain, stiffness,  and functional scores decreased significantly (P<0.001). AUSCAN pain and functional scores decreased significantly (P=0.007 and P=0.004). BPI overall pain score decreased significantly.  FSI fatigue score decreased significantly (P=0.004).  HFRDIS surge red score  decreased significantly (P=0.02). PSQI sleep quality  score was significantly improved (P<0.001).  CES-D depressive symptom score decreased significantly. | 1. The findings  suggest that tele-tai chi intervention can alleviate AI-induced joint pain and improve quality of life. It is recommended that it be rolled out to a broader  patient population.  2. Future studies should consider providing more  flexible course scheduling to meet the time needs of different patients and improve the course participation rate. | **Strengths:**  1. Remote intervention  2. Multidimensional evaluation  3. Safety: No taiji-related adverse events were reported in the study  **Limitations:**  1. Single-arm design: lacks a control group,  which may be affected  by selection bias and the placebo effect.  2. Insufficient sample diversity.  3. Course participation rate: The course participation rate of participants was only 61%.  4. Technical obstacles: Some elderly patients encounter technical difficulties when using the Zoom platform,  which affects the course. |
| Çınar, Karadakovan and Erdoğan/2021 | The study's purpose was to  determine the effects on quality of life (QoL) of a mobile phone  app-based training for supportive care of women with breast cancer who were using adjuvant endocrine hormonal therapy | **Methodology:** A randomised pre-post test design.  The study combined two models: mobile app-based patient education (1) and web-based management application (2). The mobile  app offers basic breast cancer information, a symptom diary and lifestyle advice  **Data Collection:**  1. Participant information sheet  2. FACT-ES Quality of Life Scale (FACT-ES QLS):  3. NCCN Pain Thermometer: | Power analysis was conducted, and 64 patients were required for the study: treatment group (n=31) and control group  (n=33).  While calculating  the study's power analysis, it was considered that approximately 40% of breast cancer cases in the databases of national cancer registry units received adjuvant EHT. | 1. The intervention group  significantly improved quality of life after the intervention, especially in  the sub-dimensions of physical, emotional and endocrine symptoms. Quality of life decreased in the control group.  2. Levels of distress were significantly lower in the  intervention group and increased in the control group.  3.App acceptance: Most participants found the mobile phone apps "informative and useful" | The study recommends mobile app-based training as a supportive care measure for women with breast cancer,  especially in dealing with EHT side effects and managing symptoms. Mobile apps can increase patient  satisfaction and  enhance trust and communication between patients and nurses. | **Strengths:**  1. Multidisciplinary collaboration: psychologists, dietitians, etc  2. Innovative: This is the first study in Turkey to explore the impact of app-based training on the  quality of life for women with breast cancer  **Limitations:**  1. Single-centre study: The study was conducted  at only one hospital; the participants were all smartphone patients.  2. The specific time and frequency of application use by participants were not measured.  3. Communication with  participants over the phone in the study may have impacted the results, but the effect of the telephone intervention  could not be assessed separately. |
| Smith et al./ 2023 | This study was designed to evaluate the feasibility of electronic patient-reported results  (ePROs)  collected through a smartphone app for symptom  monitoring in breast cancer patients during the first year of endocrine therapy (ET) | **Methodology： A prospective observational study**  Participants completed ePRO surveys via a smartphone app at baseline, 1 month, 3 months, 6 months and  12 months. When a symptom score exceeds a preset threshold, the system triggers an alert and provides the clinician a recommended symptom management path.  **Data Collection：**Data was  collected via a smartphone  app, and participants completed ePRO surveys that included:  1. PROMIS Scale  2. PRO-CTCAE Scale | The study included250 breast cancer patients receiving  endocrine therapy, aged 28-83 years, mostly white (73%), and most patients with stage I-II breast cancer (88%). Participants were recruited through screening  and physician referrals by Johns Hopkins University and Allegheny Health Network  (AHN). | 1. Survey completion rate: 73.2% of participants completed the baseline survey, and 69.6% completed at least one follow-up survey within 6 months, meeting the study's primary feasibility goal.  2. Symptom Alert: 31% of participants had at least one symptom alert at baseline, and 74% had at  least one symptom alert during follow-up.  3. Path implementation: Although the confirmation rate of alerts was low  (51.1%-62.1%), the  12-month ET  discontinuation rate was 11.2%, below the  preset target of 15%. | 1. Increase alert confirmation rates: future studies should consider how to increase clinician engagement.  2. Integration of Electronic Health Records (EHR)  3. Optimize follow-up time: Combining ePRO surveys with clinical visit time may improve patient  engagement and clinician response rates. | **Strengths:**  1. Real-time symptom  monitoring  2. Personalized management path  3. High completion rate  **Limitations:**  1. The sample size is small  2. English-speaking smartphone users, which may limit the generality of the results  3. EHR not integrated  4. Low confirmation rate of alerts |
| Chan et al./ 2024 | This study was  designed to explore the feasibility and effectiveness of a mobile app-based rehabilitation program called THRIVE for breast cancer survivors undergoing hormone therapy | **Methodology：**  The study used prospective  single-arm participants who  used the THRIVE mobile app and Fitbit activity tracker for a 16-week rehabilitation program. Primary assessment measures included recruitment rate,  dropout rate, compliance rate, and safety, while secondary  assessment measures included  physical activity intensity, HRQoL, psychological stress, body composition, and application satisfaction.  **Data Collection**: included a baseline assessment and an  assessment at 16 weeks. Participants recorded daily exercise and medication adherence through the "THRIVE" app and Fitbit. | The study recruited50 breast cancer survivors with a median age of 53. All participants had early-stage breast cancer, had completed surgery, adjuvant chemotherapy or radiation within the past five years, and were receiving hormone therapy. Exclusion criteria  include metastatic disease requiring  chemotherapy,  ECOG performance status ≥3, cognitive impairment or physical impairment that prevents participation in physical activity. | 1. The recruitment rate was 70.4%, and the compliance rate was 74%  for those who did not quit.  2. Physical activity intensity did not change significantly over 16weeks.  3.Cognitive function was  significantly improved  (p = 0.021).  4. Depression and anxiety scores were significantly reduced  5. All participants reported 100% medication adherence.  6. Participants are highly satisfied with the app, with98% saying they would recommend it to others. | 1. Future studies should consider prolonged intervention to  observe more significant changes in physical activity. 2. Apps should  add personalized goal setting, real-time feedback,  and social support to increase user engagement and effectiveness.  3. To enhance the generality of the results, future studies should include more patients with advanced breast cancer. | **Strengths:**  1. High engagement  2. Comprehensive assessment  3. Culturally adaptable  **Limitations:**  1. Small sample size  2. Single-center study: It may limit the wide applicability of the results.  3. Lack of control group: The study was designed as a single-arm study, the absence of a control group complicated the ability to eliminate the influence of other factors. |
| Bergq-vist,  Lunds-tröma-nd Wengs-tröm /2021 | The study's primary aim was to develop and  investigate a patient interactive digital support (an app) for patients on adjuvant endocrine breast cancer treatment. | **Methodology: A multi-stage mixed research method**  **Data Collection**: 1. Medication adherence: Record whether patients take their medication on time.  2. Physical exercise: The patient's daily physical activity time and intensity are recorded.  3. Self-care activities: Records of self-care activities undertaken by the patient, such as massage and social activities.  4. Health and quality of life: Answer 13 weekly health and quality of life questions based on the EORTC QLQ-C30 and EORTC QLQ-BR23 questionnaires.  5. System Usability Score  (SUS): At the end of the study, patients completed the SUS questionnaire to assess the application's ease of use and value. | The pilot study included 15 breast cancer patients aged 41 to 78 years who were receiving adjuvant endocrine therapy | 1. Usability: Patients found the app easy to use  2. 15 patients registered 4,251 times over a 3-month period, with a median of 262. Most registrations involved medication adherence (1,582) and physical exercise (1,125).  3. Patients answered 13 weekly questions about health and quality of life, Hot flashes, sleep disturbances, and muscle  and joint pain are the most common symptoms. 4. Medication adherence: Almost all patients took their medication on time.  5. Patients walked an average of 45 minutes per day, with a median enrollment of 45 days. | 1. Added functionality: Patients want the app to be  able to connect with other apps, such as exercise and diet logging apps, and they want to interact with other patients using the app.  2. Extension of  study time: Future studies should consider  extending the duration of the  intervention to  observe longer-term effects.  3. Personalized support: Future digital support tools should provide more personalized features | **Strengths:**  1. The application's SUS score is very high,  indicating a high degree of patient approval of its ease of use and value.  2. The study comprehensively assessed medication adherence,  physical exercise, self-  care activities, and quality of life.  **Limitations:**  1. Small sample size: It may affect the generality of the results.  2. Short study duration3. Lack of control group |
| Graetz et al./ 2018 | Improving symptom burden and medication  adherence with a web-based application (app) for postmenopausal women with hormone receptor-positive, long-term aromatase inhibitor (AI)-treated breast cancer. | **Methodology：** randomized  controlled trial (RCT).  **Intervention:** The app's functionality involves real-time reporting of symptoms (severity scores)  and medication adherence  (via the MARS-1 scale), with automated alerts sent to the healthcare team upon triggering thresholds. Alert thresholds included new symptoms, an increase in symptom severity by ≥4 points, and missing ≥2 doses of medication.  **Data Collection:**  1. Baseline data  2. Follow-up data (after 6-8 weeks): medication adherence (MMAS-4). Symptom burden (FACT-ES). | Sample size: 44 patients who completed the intervention and follow-up (56 initially enrolled, 8 excluded, and 4 dropped out midway)  Divided into two  groups: App+ Reminders group: Receive weekly SMS/email reminders using theApp to report symptoms and medication adherence. App group: Only App  access permission is provided without active notification. | 1.Weekly usage was significantly higher in the App+ reminders group  (74% vs. 38%, p<0.05).  2. Complete compliance in the App+ reminder group  reached 100% at 8 weeks, which was significantly higher than that in the App group.  3. The burden of symptoms increased more  in the App group but was not statistically significant (p=0.191). | 1. The association between long-term adherence  and survival outcomes was validated by expanding the sample size and extending follow-up (e.g., 5 years).  2. Integrate App interventions into routine clinical  workflows, with a focus on low-income and minority patients.  3. Explore other technical means, such as artificial intelligence, to  optimize symptom monitoring | **Strengths:**  1. 36.4% of low-income and 22.7% of black patients were included  **Limitations:**  1. The sample size was small: only 44 cases, the statistical power was insufficient, and the difference in symptom burden was insignificant. 2. Short-term follow-up  3. Self-report bias  4. Single centre restriction |
| Graetz et al./ 2024 | To test whether  remote monitoring of symptoms  and treatment adherence with  or without tailored text messages improves outcomes among women with breast cancer who are prescribed AET. | **Methodology:** This nonblinded, randomized clinical trial (RCT)  **Groupings:** Participants were randomly divided into three groups: 1. Application Only group 2. App plus feedback group 3. Enhanced Usual Care Group (EUC)  **Data Collection:**  **Key indicators:** 1-year AET compliance (percentage of days of compliance) recorded by electronic pill box.  **Secondary indicators:** Symptom management in medical records . App usage data (number of logins, alerts triggered). Tools: Electronic  pill kits (Wisepill RT300), standardized questionnaires (e.g. FACT-ES, SF-12). | Electronic health record screening + physician recommendation finally enrolled 304patients.  Inclusion criteria: spoken English, early-stage breast  cancer (hormone receptor-positive),  new AET prescription, mobile device and mailbox  ownership. | 1. There was no significant difference between the three groups (76.6% in the EUC group, 73.4% in the application-only group, and 70.9% in the application-plus feedback group; All P>0.05).  2.Total medical , high-cost contact (-0.40, P=0.003) and number of outpatient  visits in the application plus feedback group were significantly lower than those in the EUC group. There was no significant  difference in the application group only.  3. Other indicators, such as quality of life, symptom burden, self-efficacy, and  doctor-patient communication, showed no difference between groups. | 1. Remote  monitoring +  customized  SMS can reduce  high-cost medical contacts and should be  integrated into  routine care.  2. Future studies need to include populations with multilingual support, limited access to technology, and extended intervention duration (>6 months) | **Strengths:** RCT,  intention-to-treat analysis, 2. patients of different races and economic levels were covered  3. Innovative: It is the  first time to verify the  impact of remote monitoring + SMS on  medical utilization in early breast cancer.  4. Clinical significance  **Limitations:**  1.Sample limit  2.Time and disruption  3. Data is missing. |
| Jacobs et al./ 2021 | Develop and optimize a cognitive behavioural therapy (CBT) based telemedicine intervention (STRIDE) to improve breast cancer survivors' adherence to adjuvant endocrine therapy (AET), manage treatment-related symptoms (e.g., hot flashes, joint pain), and reduce psychological distress,ultimately improving patients' quality of life and treatment outcomes. | **Methodology**: mixed research  STRIDE intervention: Integrated cognitive behavioral therapy, relaxation training, side effect management strategies, and  peer support in 6 weeks  (subsequently adjusted to 6  group meetings +2 individual follow-up visits).  **Data Collection:**  1. Quantitative data  2. Overall satisfaction  3. Customer Satisfaction Questionnaire (CSQ)  4. Qualitative data  Semi-structured Exit Interview | Sample size: 5 breast cancer survivors (finalised).  Inclusion criteria: Women over 21 years of age with early-stage hormone receptor-positive breast cancer (stage 0-IIB) who are receiving AET treatment (starting within 1 week to 3 years). | 1.100% of participants completed all meetings and follow-up assessments.  2. Sessions scored an average of 9.55/10. With an overall satisfaction score of 3.6-4.0/4, the CSQ showed that 80% of participants felt the intervention "fully met the need."  3. Relaxation techniques,  peer support, and side effect management strategies are efficient. Improvement needs: Increased meetings (from 5to6) and extended time for side effect management. | A randomised controlled trial (RCT) was conducted to verify the effect of the intervention. | **Strengths:**  1. Theory-driven: Multi-dimensional intervention design based on the health belief model,  stress and coping theory.  2. Patient participation: Iterative optimization is used to ensure that the intervention is close to patients' actual needs through qualitative interviews and quantitative feedback.  3. Innovative: The first AET compliance intervention study to integrate remote technology  **Limitations:**  1. Sample limitations  2. Potential bias  3. Technology dependence conditions  4. Unknown long-term  effects |
| Lally et al./ 2024 | This industry-academic partnership study aimed to collect qualitative user  experience data of a prototype app with built-in peer and coach support designed to support the  management of  health behavioursand weight in women living with breast cancer. | **Methodology:** Qualitative research  Participants were asked to complete a short online survey, use an app called Healthy Habits after Cancer (HHC) for 4 weeks, and then participate in qualitative interviews  **Data Collection:**  1. Baseline survey.  2. App Usage data  3. Qualitative Interview | Eight patients,  Eight participants  eventually completed four weeks of app use and participated in interviews.  Female, ≥18 years old; Have been diagnosed with breast cancer within the last 5  years (at any stage); Have completed aggressive cancer treatment (such as  chemotherapy,  surgery, or radiation); Currently receiving oral hormone  therapy;  Smartphones with  iOS operating system. | 1. Participants felt apps were more helpful earlier in treatment, such as during chemotherapy,  because they had more time and a strong need for health information.  2. Participants wanted the app to focus more on weight management,  specifically weight changes related to breast cancer treatment and how to cope with those changes.  3. Participants generally liked the diet recording,  water intake, and exercise  4. Participants rated one-on-one coaching and group  support features highly as providing accountability and emotional support. | 1. Early intervention  2. Apps should  add more weight management content related to breast cancer,  especially dietary recommendations for treatment side effects (e.g., nausea and diarrhoea).  3. Apps should  allow users to  adjust step and  calorie goals  based on  individual  circumstances  to increase  challenge and  motivation.  4. Strengthen social support | **Strengths:**  Through qualitative interviews, the study provided an in-depth understanding of the experience of breast cancer patients. **Limitations:**  1. Small study sample size (8 participants)  2. The interview was conducted by one of Noom's researchers and may introduce certain biases |
| Ahlste-dt Ka-rlsson et al. /2022 | This study was  designed to explore the feasibility and patient acceptance of an individualized support program (RESPECT intervention) for breast cancer patients undergoing endocrine  therapy(ET) | Methodology:  Randomised pre-post  controlled study  The intervention group received personalized educational materials, learning plans, and personalized reminder letters, and the intervention could be delivered over 12 weeks via telephone or digital follow-up. The control group received routine care.  Data Collection:  1. The General Self-Efficacy Scale (GSE)  2. Patient-perspective Quality of Care Questionnaire (QPP  3. Memorial Symptom Assessment Scale (MSAS) | The study enrolled 66 eligible breast  cancer patients between September 2020 and June 2021; ultimately, 41 patients participated. There were 21 participants in the intervention group and 20 in the control group. Participants were  women over 18 years of age diagnosed with breast cancer and  treated with ET, excluding patients  receiving adjuvant chemotherapy. | 1. 86% of patients in the  intervention group completed the intervention and 3-month post-questionnaire, and attendance at telephone follow-up was 90%. The intervention nurse strictly  followed the intervention  protocol.  2. There were no  significant differences in  self-efficacy and symptom  management between the  intervention and control groups.  3. The control group scored higher on quality of care than the intervention group.  4. Patients preferred telephone follow-up rather than face-to-face meetings. | 1. Telephone follow-up is the preferred intervention for  patients and may be considered for continued use in future studies.  2. An intervention duration of 12  weeks is appropriate, but an additional follow-up after 6months may be considered in the future to help patients better cope with the long-term side effects of ET. 3.The self-efficacy baseline was higher in the intervention group and the control group | Strengths：  1. Based on self-care theories as well as person-centred care models  2. Methodological rigor, following the CONSORT 2010 statement  3. High completion rate  Limitations：  1. Small sample size  2. Before and after controlled trials, not RCTS  3. Short intervention time  4. Limitations of the self-efficacy scale |
| Park et al. /  2022 | This study aimed to evaluate the  effect of a reminder  intervention—a smart pill bottle paired with the Pillsy mobile  application— on medication adherence,  medication self-efficacy, and depression among breast cancer survivors who  were undergoing oral antiestrogen therapy. | **Methodology:**  Randomized controlled trial.  Intervention: The experimental group used a smart pill bottle (Pillsy) for 4 weeks. The pill bottle reminds you to take medicine by beeping and flashing lights, and it synchronises the opening time with the mobile app. The "help" reminder function was supported  **Data Collection：**  Medication compliance: calculated by bottle opening records (experimental group) and medication logs (control group).  Self-efficacy: A 5-point Likert drug self-efficacy scalewas used (8 items,  Cronbach's α=0.71).  Depression  Structured questionnaires and electronic records of smart pill bottles. | Grouping: 61 participants were randomly assigned to the experimental group (31 with smart pill bottles) and the control group (30 with usual care).  Final sample: 57  completed follow-ups (30 in the experimental group and 27 in the control group) | 1. Medication compliance: The experimental group's compliance was significantly higher than that of the control group (97.3% vs. 88.3%, P=0.004).  2. Self-efficacy: The experimental group's post-test scores significantly improved (P=0.004), while the control group's scores did not significantly change.  3. Depression: There was no significant difference between the two groups  (P=0.057). | 1.Smart pill bottles can be an effective tool for improving medication compliance,  especially for patients who miss medication due to forgetfulness.  2. Psychological support interventions are needed to mitigate the effects of depression on compliance; Long-term studies demonstrate sustained effects of the intervention; Explore the role of family members as "helpers." | **Strengths:**  1. For the first time, the intervention effect of smart pill bottles has been validated in breast cancer survivors  2. Combine real-time alerts, help notifications, and mobile app tracking  **Limitations:**  1. Single-centre study with a small sample size  2. Only 4 weeks; long-term effects cannot be assessed  3. Bottle opening records may overestimate compliance |
| Takada et al./ 2022 | The present study aims to evaluate a novel coping skills training intervention protocol  (CST-AET) for  improving women’s abilities to adhere to AET and reduce how symptoms  interfere with quality of life and daily activities. | **Methodology:** Prospective study  1. Patients install the PHR app on their smartphone or  tablet  2. Patients are asked to record their daily medication and symptoms during the one-month study period  **Data Collection:**  1. Quality of life was assessed using the Functional Assessment Cancer Therapy-Breast Cancer  (FACT-B) questionnaire  2. Assessed twice before the start of treatment and one  month later  3. Collection of daily medication and symptom data recorded by patients on the PHR app  4. Questionnaire at the end of the study to collect patients' opinions on the PHR app | 14 breast cancer  patients receiving  adjuvant hormone therapy  1. Treated at Showa University Hospital  2. Age 20 years or older  3. All patients were hormone receptor-positive | 1.All patients were able to use the PHR app without compromising their quality of life - 79% of patients had complete documentation of PROs  2. Approximately 70% of patients want to use the PHR app to communicate with healthcare professionals in the future  3.90% of patients who found it challenging to communicate with healthcare professionals wanted to use the PHR app  4. Some patients want to use the PHR app as a medication reminder tool | 1. Establish a system for medical staff to regularly monitor PROs recorded on the PHR app  2. Integrate the PHR app system into the electronic medical record  system  3. Healthcare professionals should validate patients' daily medication records reported on the PHR app and provid recommendatio-ns | **Strengths:**  1.Prospective study design  2. Explored the potential of the PHR app in adjuvant hormone therapy  3. Provided real-world patient experience and feedback on the use of the PHR app  **Limitations:**  1. Small sample size  2. Short study period  (only one month)  3. Single-arm study, lack of control group |
| Walsh et al./ 2024 | This study examined whether changes in coping and self‐efﬁcacy mediated intervention effects on anxiety,  depression, QOL,and symptom distress. | **Methodology:**  A randomised controlled trial. Patients completed self-report scales assessing anxiety, depression, quality of life, symptom distress, coping ability, and self-efficacy at  baseline and 24-week follow-up. The study used mediation regression modelling to test whether coping ability and self-efficacy changes mediated the intervention effect.  **Data Collection:**  1. Hospital Anxiety and Depression Scale  2. Functional Assessment of Cancer Therapy-Breast Cancer Scale (FACT-B)  3. Symptom Scale (BCPT)  4. Measure of Current Status-Section A (MOCS-A) | Women (N = 100) were recruited between 10/2019  and 06/2021 from Massachusetts General Hospital  and were randomised to STRIDE or the medication monitoring control group. | 1. Improvements in coping mediated the effect of the STRIDE intervention on anxiety symptoms  2. Improvements in coping mediated the effect of the STRIDE intervention on depressive symptoms  3. Improvements in coping mediated the effect of the STRIDE intervention on quality of life, coping skills did not mediate the  impact of the STRIDE intervention on symptom distress  5. Changes in self-efficacy did not mediate the intervention effect of STRIDE on any outcome  variable | 1. Further optimise the STRIDE intervention to  enhance the content and intensity of coping skills training  2. Future studies could explore other potential  Mediating  mechanisms, such as quality of patient-physician communication,  social support and other factors | **Strengths:**  1. Uses theory-driven mediation analyses that  provide insight into the  intervention's mechanism of action  2. Focuses on clinically  relevant outcome variables  **Limitations:**  1. High homogeneity of the sample  2. relatively small sample size  3. Reliance on self-report scales, possible reporting bias  4. Long-term effects were not considered  5. Other potential mediating variables were not explored |
| Moug-alian  et al./ 2017 | This study evaluates a bidirectional text messaging application called BETA-Text,  which monitors  adherence to endocrine therapy and patient-reported outcomes in breast cancer patients. | **Methodology:** A pilot study  The intervention consisted of text messages that patients responded to over a 3-month periodIf patients report a worrisome reaction, this triggers a return phone call  from the clinic nurse.  **Data Collection:**  1. Patient-reported data collected through the BETA-Text application  2. Patient-completed self-report surveys  3. Medical record review  4. Historical control group data | The study recruited 100 patients with stage I-III hormone receptor-positive breast cancer patients。 | 1. 98%of patients reported that the intervention was easy to use, and 96% found it helpful in taking their medication.  2. 93% of patients who continued treatment took ≥80% of their medication.  3. adverse reactions reported:2.4% reported hot flushes,53.1% reported  Joint pain,a34.7% reported vaginal symptoms  4. Incidence of adverse reactions: Adverse reactions to endocrine therapy detected by this SMS method were more common than those reported in clinical trials. | 1. BETA-Text can be an effective tool for real-time monitoring of patient adherence and identification of adverse events.  2. this approach may improve patient engagement and patient-provider  communication.  3. further testing of the effectiveness of BETA-Text in larger randomised controlled trials should be considered. | **Strengths:**  1. BETA-Text is the first adherence improvement tool that allows structured patient communication outside the clinic.  2. high acceptance  3. real-time data collection  4.Simultaneously, multiple adherence-related factors, are addressed.  **Limitations:**  1. Small sample size  2.use of historical controls may introduce bias  3.adherence measures relied on self-report and may not be as accurate as objective measures.  4.the short duration of the study (3 months) may not reflect long-term adherence patterns. |
| Okuya-ma et al./  2024 | This study aims to evaluate the  impact of symptom monitoring using the electronic Patient Reported Outcomes (ePRO) application on health-related quality of life  (HRQoL) in postmenopausal breast cancer patients receiving adjuvant endocrine therapy. | **Methodology:** Single-centre, open-label, randomised controlled trial  1. The ONC group reported 5 symptoms per week via the app for 3 months  2. The primary endpoint was HRQoL at 3 months assessed using FACT-B  **Data Collection:**  1. Baseline characteristics were obtained from medical records  2.HRQoL assessed using FACT-B  3. Patient-provider communication quality assessed using EORTC QLQ-COMU26  4. ONC group collects PRO-CTCAE symptom reports via an app | 125 patients (61 in the ONC group and 64 in the control group)  Inclusion criteria: ≥20 years of age, hormone receptor-positive breast  cancer,  postmenopausal, planned use of aromatase inhibitors | 1. ePRO monitoring did not significantly improve  HRQoL (FACT-B total score difference: -1.55, 95% CI: -5.91, 2.81)  2. PRO-CTCAE response  rate in the ONC group remained above 70% in the first 10 weeks  3. arthralgia and insomnia were the most frequently  reported symptoms  4. COMU26 scores showed a downward trend in both groups but were slightly higher in the ONC group | 1. Effective synchronised interventions for reported symptoms need to be developed  2. future studies should evaluate the impact of appropriate patient complaint-based interventions on symptom improvement and treatment adherence  3. further validate the potential of ePRO monitoring as a patient-provider  communication tool | **Strengths:**  1. Randomised controlled trial  2. results assessed using validated tools  3. high response rates and zero missing items  demonstrate the feasibility **Limitations:**  1. The intervention period is only 3 months  2. the intervention accurately captured patients' symptoms but did not provide effective symptom-specific interventions  3. small sample size |
| Brett, Boult-on and Watson/2018 | To explore, develop and pilot a patient-centred e-health app to  support and motivate women taking adjuvant  endocrine therapy (AET) after breast cancer treatment. | **Methodology:** Qualitative approach  Three phases:  1. Development phase (focus groups and interviews)  2. Usability testing  3. Pilot testing  **Data Collection**: Through focus groups, interviews, and usability assessments | 1. Women who had completed active treatment for early-stage breast cancer (I-IIIA)  - 18 years or older  - Able to provide  informed consent  - For the pilot phase: owned a smartphone, tablet, or computer  - Recruitment through UK charity Breast Cancer Care and Independent Patient Cancer Voices | Specific Findings on App Sections  1. Side-effects diary: Most found it helpful for recording and managing side-effects  2. Information section: Generally found useful and easy to understand  3. Online forum: Mixed views on usefulness  4. Prescription reminder: Potential usefulness recognized  5. Useful links | 1. Further development and evaluation of the app needed  2. Assess whether the app supports women and improves adherence to AET  3. Consider incorporating colourful icons to make the interface more user-friendly  4. Address technical issues (e.g., forum access) in future versions | **Strengths:**  1. Original data on patients' views of innovative technology  2. Pragmatic 'real-life' exploration  3. Low-cost resource (used on personal devices)  4. Patient-centred development process  Limitations:  1. Convenience sample  2. Limited funds prevented the development of the more technically advanced app  3. Small sample size for pilot testing |
| Harbe-ck et al./  2023 | To assess the safety impact of the interactive autonomous eHealth support  System  (CANKADO PRO-React) on patients receiving pabocinib and endocrine therapy,  particularly on the risk of serious adverse  events (SAEs). | **Methodology:**  Multicentre, randomised, parallel-group, phase IV clinical trial  **Data Collection:**  1. Collect patient-reported outcome (PRO) data  2. Record adverse events (AE) and serious adverse events (SAE)  3. Classify AEs and SAEs  using the MedDRA coding  system  4. Collect treatment characterisation data such as relative dose intensity, delays,  interruptions and dose reduction rates | Safety group: 479 patients  CANKADO-active group: 318  CANKADO-inform group: 161  Includes all participants who received at least one dose of palbociclib | 1. The risk of the first SAE was significantly lower in the CANKADO-active group  2. at 24 months, the incidence of SAE was 22.9% in the CANKADO-active group and 30.3% in the CANKADO-inform group  3. the overall distribution of AE was similar between the two groups  4. AE-related dose reductions affected approximately 20% of patients | 1. consider the use of interactive autonomous eHealth support systems in oral oncology treatment for patients with advanced and  Metastatic  HR+/HER2- breast cancer  2. further investigate the  potential impact of eHealth support on patient adherence and long-term outcomes  3. development of artificial intelligence-based event prediction and early warning algorithms to further of | **Strengths:**  1. first randomised prospective trial to demonstrate a substantial effect of interactive autonomous eHealth support on SAE risk  2. A large sample size  3. uses competing risk  analyses  4. Provide detailed safety data  **Limitations:**  1. The study stopped early due to the COVID-19 pandemic  2. study not designed to detect a modest survival advantage |
| Richar-dson  et al./ 2021 | To assess and describe patient-reported outcomes (PROs)in female patients with HR+/HER2- locally advanced/ unresectable or metastatic breast cancer(aBC/mBC) treated with palbociclib combination therapy in a real-world setting in the United States. | **Methodology:**  Prospective, non-interventional,  multi-centre longitudinal study  **Data Collection:**  1. PRO data: collected at daily, weekly and weekly intervals via mobile application  2. Medical information: collected from medical records at baseline and the end of the 6-month follow-up period - PROs collected include  (SF-12, CES-D-10, Mood,Pain, Fatigue, Impact of aBC/mBC or its treatment on all aspects of life, Overall health rating, Quality of life) | convenience sample  139 evaluable patients recruited from 25 participating centres | 1. Patient-reported levels of pain and fatigue were low and remained stable throughout treatment  2. General health status  (SF-12) remained consistent throughout treatment, in line with published standards for cancer patients  3. Depressive symptoms  (CES-D-10) were low  4. patients, on average,  reported neutral or positive moods | The study's results provide  valuable data for treatment discussions between patients and physicians  and medical decision-making. These findings  may help physicians better understand the impact of palbociclib combination therapy on patients' quality of life and thus make more informed treatment decisions. | **Strengths:**  1. Collects PRO data using an innovative mobile application,  2. Incorporates multiple PRO measurement tools. **Limitations:**  1. Participating centres  are a convenience sample  2. Only 139 evaluable  patients were recruited,  falling short of the target 300.  3. Completeness and accuracy of self-reported results may be problematic.  4. Some patients may have withdrawn early from the study due to disease progression or declining health status |
| Hersh-man  et al./  2020 | The purpose of this study is to evaluate whether text message reminders reduce the likelihood of premature discontinuation of adjuvant aromatase inhibitor (AI)  therapy in women with early-stage breast cancer. | **Methodology:**  Multi-centre randomized controlled trial  Duration: 36 months  Intervention  - TM group: receive educational text messages twice a week  - No-TM group: usual care, not receiving text messages  **Data Collection:**  1. Assessments at baseline and every 3 months  2. Collection of patient-reported outcomes and urine samples  3. Detect AIs and their metabolites in urine using liquid chromatography/ tandem mass spectrometry or gas chromatography/mass spectrometry  4. Record patient self-reported and provider-reported AI discontinuations | A total of 724 patients were included from 40  institutions  Eligible patients: 702 (348 TM group, 354 No-TM group) | 1. Primary outcome: at 36 months, there was no significant difference in adherence failure rates between the TM and No-TM groups  TM group: 81.9%  No-TM group: 85.6%  Risk ratio (HR) = 0.89  (95% CI: 0.76-1.05), p= 0.18  2. Secondary outcomes:  Patient self-reported discontinuation rate: 10.4% in the TM group vs 10.3%in the No-TM group  Facility-reported discontinuation rate: 21.9% in the TM group vs. 18.9% in the No-TM group. | 1. future interventions should consider more individualised and sustained behavioural interventions  2. improved two-way communication  may be needed to intervene early to prevent discontinuation of medication  3. attention should be given to symptom management and support to improve long-term adherence  4. future studies may consider interventions for patients with known adherence problems  5. more effective educational methods and communication strategies should be explored to increase patient awareness of the importance of AI treatment | **Strengths:**  1. large sample size  2. Long-term follow-up  3. multi-centre study  4. multiple methods were used to assess adherence  **Limitations:**  1. Inability to distinguish between missed appointments and actual medication non-adherence  2. SMS reminders can lead to ‘alert fatigue’ and information overload  3. interventions do not target specific causes of non-adherence  4. self-reported and provider-reported adherence may be biased |
| Aurell,Haidar and Giglio /2024 | This study assesses adherence to endocrine therapy in breast cancer patients in western Sweden, explores the factors influencing adherence, and  examines the effect of different follow-up protocols on adherence. | **Methodology:**  A retrospective study assessed patient adherence to endocrine therapy by analysing electronic medical  record data.  **Data Collection:**  The study collected the following data from electronic medical records:  Demographic data,  Clinical data,  Pathological data,  Endocrine treatment utilisation,  Reasons for change in treatment, Side effects,  Frequency of prescription updates,  Breast cancer recurrence and death | A final total of 358 patients was  included | 1. Endocrine therapy adherence was generally high, with medication adherence rates of 72.0% (tamoxifen) and 75.7%  (aromatase inhibitors) at 4 years and beyond  2. Adherence was better in follow-up regimen B  (less nurse-initiated contact) than regimen A  3. Treatment changes due to side effects were more  frequent in the aromatase  inhibitor group (24.3% vs 9.9%, P < 0.0001)  4. Experiencing treatment  side effects was an independent factor influencing adherence  5. Tumour characteristics  and adjuvant treatment were not associated with  adherence | 1. Randomised controlled trials  are needed to determine the optimal follow-up protocol in adjuvant breast  cancer treatment  2. More information and support may be needed to improve adherence for patients using hormone replacement therapy  3. Attention should be paid to patients' treatment side effects,and appropriate management strategies should be provided to  Improve adherence | **Strengths**. Ability to track almost all patient  follow-ups  2. Analysed changes in  endocrine therapy over time  3. provides new insights into the impact of different follow-up regimens on adherence  4. Findings may have important implications for improving the management of long-term treatment for breast cancer patients  **Limitations:**  1. fixed sample size  2. Patients were not recorded as picking up medication from the pharmacy, which may affect the accuracy of adherence assessments  3. Breast cancer-specific survival may have been overestimated**.** |
| Tan et al./  2020 | To assess the effect of short message service (SMS)  reminders on adherence to adjuvant aromatase inhibitors (AIs) and serum hormone levels in female patients with breast cancer. | **Methodology:**  Open-label, multi-centre,  prospective randomised  controlled trial  - Primary outcome indicator: medication adherence  - Secondary outcome indicator: serum hormone levels (androstenedione,  oestradiol, oestrone)  **Data Collection:**  1. Medication adherence:  Assessed at baseline, 6 months, and 1 year using the Simplified Medication Adherence Questionnaire  (SMAQ)  Patient logs and pill counts were used to validate the accuracy of the SMAQ.  2. Serum hormone levels:  Measured at baseline and 1 year | A total of 244 patients were recruited (123 in the SMS group and 121 in the standard care group) | 1. Medication adherence:  At 6 months, adherence  was significantly higher in the SMS group than in the standard care group (72.4% vs 59.5%, p=0.034)  At 1 year, the difference  between the two groups was not significant (68.9% vs 65.8%, p=0.617)  Mixed-effects logistic regression analysis showed an adherence advantage ratio of 2.35 (95% CI: 1.01-5.49, p=0.048) for the SMS group over the 1-yearperiod | 1. consider  incorporating  SMS reminder  services into  routine care  2. future research could tailor SMS interventions to  patient preferences,  3. explore multi-component intervention strategies combining educational,  attitudinal and technological aspects to achieve long-term behavioural change | **Strengths:**  1. Randomised controlled trial design  2. multi-centre studies  3. use of multiple methods to assess medication adherence  4. long-term follow-up  5. high acceptability  **Limitations:**  1. approximately 20% of patients in the standard  care group used their own reminders  2. Patient logs may inadvertently serve as reminders  3. self-reported adherence may not be entirely accurate |
| Neuneret al./  2022 | To assess the feasibility of a  novel, clinical pharmacist-led intervention targeting symptom and medication management designed to improve adherence to AET in early nonadherent patients. | **Methodology：**  Pilot study on the feasibility of a single-arm  1. 6-month intervention  2. Use of electronic symptom monitoring and guideline-based symptom management  3. Intervention by clinical pharmacists outside of scheduled office visits  **Data Collection：**  1. Symptom reporting  2. Patient-reported outcomes (PROs)  3. Pharmacist time and intervention content  4. Adherence: use of Surescripts electronic all-payer, all-pharmacy claims database for PDC calculation | Sample size: 20 patients enrolled;19 completed the initial assessment, and 18 completed all final study procedures | 1. Feasibility:  - 18/19 participants completed all final study  procedures  - 14 completed all assessments, and no patient missed more than 3assessments  2. Symptom reporting:  - All 18 participants reported at least 1 symptom type  - Most participants tried the pharmacist's recommendations  3. PRO Improvement:  - Improved patient-reported indicators of physical,  mental and social health and self-efficacy  4. Improved compliance. | 1. Larger, longer-term randomized controlled trials to determine the effectiveness and cost of interventions  2. Expansion of the 1-hour training course  and provision of training information for oncology providers  3. Consider  routing PRO assessments through the telephone interactive voice recording system to improve accessibility  4. Track potential costs and training needs for implementing interventions in  future studies | **Strengths：**  1. Innovative intervention approach  2. Targeting high-risk non-adherent populations  3. Utilizing existing clinical pharmacist resources  **Limitations：**  1. Small sample size,  single-centre, before-and-after design pilot study  2. Relatively short follow-up period  3. Use of pharmacy fill information may miss some non-adherent patients  4. Informal training of the research team may have influenced the results. |
| Advaniet al./ 2017 | To assess whether early intervention reduces sexual  dysfunction within the first  year of aromatase inhibitor treatment. A secondary objective was to compare the effects of two vaginal moisturizers. | **Methodology：** Randomized  controlled trial  Randomized into three groups:  1. Routine care group  2. Active treatment group H (hyaluronic acid-based vaginal moisturizers)  3. Active treatment group P (probiotic vaginal moisturizer)  **Data Collection:**  Questionnaires were completed at baseline, 6 months and 12 months:  - Female Sexual Function Index (FSFI)  - Menopausal Sexual Interest Questionnaire (MSIQ)  - Female Sexual Distress Scale-Revised (FSDS-R)  - Menopause Symptom Scale | Total sample size: 57  Follow-up data providers:49 (86%)  Average age: 59  years  77% non-Hispanic white | 1. Impaired sexual functioning at baseline but stabilized in all groups over 12 months  2. At 6 months, the combined active treatment group compared to the usual care group:  - Less painful intercourse  (P = 0.07)  - Less sexual distress  (p = 0.02)  3. At 6 months, improvement in total FSFI score was significantly better in the active treatment group H than in the active treatment group P (p = 0.04) | 1. Sexual Counselling Helps Women  Maintain Stable Sexual Function During Aromatase Inhibitor Use  2. Proactive interventions lead to better outcomes at 6 months  3. More research is needed on the role of preventive counselling in maintaining sexual functioning during treatment with aromatase  inhibitors | Strengths:  1. Randomized controlled designs enhance the internal validity of studies  2. Assessment of sexual functioning and related  symptoms using multiple standardized questionnaires  3. Attention was given to a clinically essential but often neglected issue  Limitations:  1. Small sample size (57 participants)  2. The nature of the experiment is a pilot trial.  3. Relatively short follow-up period (12 months)  4. Failure to detail reasons for dropout (86%provided follow-up data) |
| Harrel-l,  Fabbri and Levy/  2017 | Analyse the practice of adjuvant endocrine therapy for breast cancer patients in the real world | **Methodology:**  observational studies  Data source:  Electronic Health Record  (EHR) data from Vanderbilt University Medical Centre.  **Data Collection：**  1. Drug events and timestamps  2. ICD-9 diagnostic codes  3. Death and recurrence information | This study utilized a full-sample analysis, which included all 1,587 patients who met the inclusion criteria, without a specific sampling  process. | 1. About 48% of patients do not complete the recommended 5 years of treatment  2. 52% of patients changed endocrine therapy drugs during treatment  3. Patients who changed medications were more likely to complete 5 years of treatment (60% vs. 37%)  4. Patients who completed 5 years of treatment had a lower relapse rate  (3.4% vs. 8.0%)  5. Higher rates of arthritis and hot flashes in patients who discontinued or changed medications  6. Switching from AIs (aromatase inhibitors) may be associated with adverse effects, mainly in the early stages of treatment. | 1. Physicians should closely monitor patients' adherence to treatment,  especially in the early stages of  treatment.  2. Considering the patient's age and menopausal status to select the  appropriate drug  3. Active management of  adverse effects to improve patient  compliance with treatment  4. Utilizing electronic health record systems to track and analyze long-term patient care | **Strengths：**  1. Utilizing real-world data  2. Large sample size  3. Integration of data from electronic health records and tumour registry systems improves data comprehensiveness  4. Innovative use of EHR data **Limitations:**  1. Potential lack of completeness of eHealth record data  2. Recurrence and mortality rates may be  underestimated  3. ICD codes for adverse reactions |
| Yin et al./  2018 | To determine the extent to which messages sent by breast cancer patients through an online portal can indicate their potential cessation of hormone therapy | **Methodology:**  retrospective research design within the observational method.  **Data Collection:**  1. Source: Vanderbilt University Medical Centre’s de-identified electronic medical record.  2. Time span: 12 years  3. Data type:  - Patient Portal News  - Electronic medical record  information | 1. Sample size: 1106 breast cancer patients  2. Inclusion criteria:  - Diagnosis of AJCC Abstract Stage I through III invasive breast cancer  - Prescribed hormone therapy drugs  - Started treatment after the end of 2005 | 1. Messaging rate over time (risk ratio HR = 1.373, p = 0.002)  2. Mention of side effects (HR = 1.214, p = 0.006)  3. Surgery-related themes (HR = 1.170, p = 0.034)  These factors are associated with an increased risk of early discontinuation. In contrast:  1. Seek professional advice (HR = 0.766, p = 0.002)  2. Expression of gratitude to health care providers  (HR = 0.872, p = 0.044)  3. Reference to drugs used to treat side effects (HR = 0.807, p = 0.013) | 1. Using patient-generated content to study health-related  behaviours  2. Development of an automated alert system to monitor patient messages  3. Designing interventions to  help patients complete long-term treatment programs | **Strengths:**  1. Use of data-driven methods  2. Combining patient-generated information and electronic medical record data  3. Provides a new method **Limitations:**  1. Single-agency populations  2. Failure to differentiate between counselled and voluntary drug withdrawal  3. Determination of discontinuation events based on medication history recorded by the EMR system, with possible monumental time bias |
| Nathanet al./ 2023 | To assess the feasibility of the online behavioural intervention 'Finding My Way' for patients with early-stage hormone receptor-positive breast  cancer who are at risk of anti-estrogen therapy (AET)  noncompliance  with psychological and menopausal symptoms. | **Methodology:**  Mixed Method  **Data Collection:**  1. Baseline characteristics: demographic information, cancer treatments  2. Questionnaire on psychological symptoms:  - Patient Health Questionnaire (PHQ-8)  - Soma Sensory Amplification Scale (SSAS)  - Anxiety Sensitivity Index (ASI-3)  3. Questionnaire on menopausal symptoms  4. Intervention in utilization data: number of logins, duration, page views, etc.  5. Semi-structured interviews | Sample size: 10 participants  1. Early hormone  receptor-positive breast cancer  (stage 0-2)  - Inclusion criteria:  2. Planning to start AET treatment  3. Meets criteria for psychological or menopausal symptoms:  - ASI-3 > 10  - SSAS > 20  - PHQ-8 > 4  - ISI > 7  - > 2 hot flashes per 24 hours | **1. Feasibility:**  - Demand: 91% of eligible patients agree to participate  - Retention rate: 70% completion of all 6 modules (7/10)  - Participation:  - Median duration per login: 19.3 minutes  - Median number of registrations: 16  - Median completion time: 10.2 weeks  **2. Acceptability:**  - Participants found the intervention helpful and universally applicable  - Video and relaxation strategies are considered an advantage  - Recommends early implementation and increased AET-related information | 1. Consider  Providing  interventions at an  early stage of  diagnosis  2. Add specific  information on  AET mechanisms and side effects  3. Conducting larger studies to assess the clinical effectiveness of  interventions | **Strengths:**  1. Preventive interventions for patients at risk of AET non-adherence  2. Comprehensive interventions combining  psychological and menopausal symptoms  3. Quantitative and qualitative data were collected to provide a comprehensive feasibility assessment  **Limitations:**  1. Small sample size  2. Limited representation of white  3. Acceptability data are only from participants who completed the intervention and may be biased  4. The actual impact of the intervention on AET adherence was not assessed |
| Wheel-er  et al./  2023 | To assess the feasibility,  acceptability, and utility of a Motivational Interviewing (MI)-based counselling intervention to improve adherence to endocrine therapy (ET) in breast  cancer patients | **Methodology:**  Single-arm pilot study  - Motivational Interviewing (MI) intervention over 12 months  **Data Collection:**  1. Baseline survey  2. 6- and 12-month follow-up surveys  3. Monitoring Medication Adherence Using Electronic Medication Event Monitoring System (MEMS) Caps and  Bottles  4. Patient-reported satisfaction assessment | Recruitment of participants at one large academic medical centre and four community sites | 1. Feasibility and acceptability:  - 97% of participants completed at least 3 MI sessions  - 83% completed at least four sessions  - High participant retention and satisfaction, especially among black women  2. - Self-reported adherence rate at 12 months was 88% (100% for black females, 81% for non-black females)  - Using MEMS cap data, most women achieved 80%adherence days, with higher adherence among black women | 1. Further develop and test MI counselling  supports to reduce racial disparities in ET adherence  2. Evaluating the impact of interventions on racially disparate outcomes in HR+ breast cancer in larger trials  3. Consider incorporating MI counselling into  routine care for breast cancer survivors | **Strengths:**  1. Development and pilot testing of the first MI-based oral medication adherence intervention for cancer patients  2. Focus on the specific needs of black and young breast cancer patients.  3. Measuring medication adherence using multiple methods  **Limitations:**  1. Small sample size  2. Participants are self-selected to participate in the study  3. Measuring drug adherence remains challenging  4. Differences between short-term self-reporting and long-term MEMS observations may lead to biased estimates of adherence |
| Kruko-wski et al./ 2024 | This exploratory analysis revealed a positive association between engagement with a remote symptom- monitoring app  and medication adherence in breast cancer survivors undergoing AET | **Methodology: RCT**  During the six-month intervention period,  participants assigned to the  app or app + feedback conditions received a weekly text message prompting them to report their AET adherence (0 - 7 days in the past week) and relevant side effects in the app. The nursing team was required to respond to these alarms within 48 hours.  **Data Collection:**  1.App Satisfaction  2.App Use  3. AET Adherence  4. Symptom Management | Participants  (N=198) were randomly assigned to the app (n=96) or app 1 feedback conditions  (n=102); and  82.3% completed  the 6-month survey (n=163).  English women  with prescription  AET. All  participants had  mobile devices  with data plans  and email  addresses. | 1. Participants with high  app usage rates were more likely to adhere to AET  treatment (90.3%).  2. The reported symptoms and compliance led to an average of 4.2 alerts, and  the population with lower educational attainment  generated significantly  fewer alerts in the  application.  3. Most participants  (81.0%) preferred to  receive text messages  weekly or more frequently, and 61.0% indicated that  the feedback messages  were useful. | This indicates that personalized feedback information may be a strategy worth testing in future research. | **Strengths:**  The study design is  robust, with  randomization and  allocation concealment  ensuring minimal bias.  The analysis utilizes  intention-to-treat and  addresses losses to  ensure effective follow-up. The results are  comprehensively reported.  **Limitations:**  The lack of blinding for participants and  researchers is a  significant limitation,  which may lead to  biases in outcome  assessment and intervention  implementation. This lack of cost assessment limits wider application and the decision - making  process in resource –  constrained settings. |

References

25. Krok-Schoen JL, Naughton MJ, Young GS, et al. Increasing Adherence to Adjuvant Hormone Therapy Among Patients With Breast Cancer: A Smart Phone App-Based Pilot Study. *Cancer Control*. 2019;26(1):1073274819883287. doi:10.1177/1073274819883287

51. Jacobs JM, Post K, Massad K, et al. A telehealth intervention for symptom management, distress, and adherence to adjuvant endocrine therapy: A randomized controlled trial. *Cancer*. 2022;128(19):3541-3551. doi:10.1002/cncr.34409

38. Gomaa S, West C, Lopez AM, et al. A Telehealth-Delivered Tai Chi Intervention (TaiChi4Joint) for Managing Aromatase Inhibitor–Induced Arthralgia in Patients With Breast Cancer During COVID-19: Longitudinal Pilot Study. *JMIR Form Res*. 2022;6(6):e34995. doi:10.2196/34995

57. Çınar D, Karadakovan A, Erdoğan AP. Effect of mobile phone app-based training on the quality of life for women with breast cancer. *European Journal of Oncology Nursing*. 2021;52:101960. doi:10.1016/j.ejon.2021.101960

34. Smith KL, Tsai HL, Lim D, et al. Feasibility of Symptom Monitoring During the First Year of Endocrine Therapy for Early Breast Cancer Using Patient-Reported Outcomes Collected via Smartphone App. *JCO Oncology Practice*. 2023;19(11):981-989. doi:10.1200/OP.23.00038

39. Chan WL, Wong YL, Tai YL, et al. Digital Rehabilitation Program for Breast Cancer Survivors on Adjuvant Hormonal Therapy: A Feasibility Study. *Cancers*. 2024;16(23):4084. doi:10.3390/cancers16234084

29. Bergqvist J, Lundström S, Wengström Y. Patient interactive digital support for women with adjuvant endocrine therapy in order to increase compliance and quality of life. *Support Care Cancer*. 2021;29(1):491-497. doi:10.1007/s00520-020-05476-z

46. Graetz I, McKillop CN, Stepanski E, Vidal GA, Anderson JN, Schwartzberg LS. Use of a web-based app to improve breast cancer symptom management and adherence for aromatase inhibitors: a randomized controlled feasibility trial. *J Cancer Surviv*. 2018;12(4):431-440. doi:10.1007/s11764-018-0682-z

40. Graetz I, Hu X, Kocak M, et al. Remote Monitoring App for Endocrine Therapy Adherence Among Patients With Early-Stage Breast Cancer: A Randomized Clinical Trial. *JAMA Netw Open*. 2024;7(6):e2417873. doi:10.1001/jamanetworkopen.2024.17873

37. Jacobs JM, Walsh EA, Rapoport CS, et al. Development and Refinement of a Telehealth Intervention for Symptom Management, Distress, and Adherence to Adjuvant Endocrine Therapy after Breast Cancer. *J Clin Psychol Med Settings*. 2021;28(3):603-618. doi:10.1007/s10880-020-09750-4

26. Lally P, May CN, Mitchell ES, McCallum M, Michaelides A, Fisher A. Prototype of an App Designed to Support Self-Management for Health Behaviors and Weight in Women Living With Breast Cancer: Qualitative User Experience Study. *JMIR Cancer*. 2024;10:e48170. doi:10.2196/48170

53. Ahlstedt Karlsson S, Henoch I, Olofsson Bagge R, Wallengren C. Person-centred support programme (RESPECT intervention) for women with breast cancer treated with endocrine therapy: a feasibility study. *BMJ Open*. 2022;12(10):e060946. doi:10.1136/bmjopen-2022-060946

48. Park HR, Kang HS, Kim SH, Singh-Carlson S. Effect of a Smart Pill Bottle Reminder Intervention on Medication Adherence, Self-efficacy, and Depression in Breast Cancer Survivors. *Cancer Nurs*. 2022;45(6):E874-E882. doi:10.1097/NCC.0000000000001030

31. Takada F, Okuyama H, Nakamura S, Fujita K ichi. Application of Personal Health Record in Enhancing the Quality of Life in Patients With Breast Cancer Who Received Adjuvant Hormonal Therapy. *EJBH*. 2022;18(2):155-162. doi:10.4274/ejbh.galenos.2022.2021-12-2

41. Walsh LE, Dunderdale L, Horick N, Temel JS, Greer JA, Jacobs JM. Intervention‐Related Changes in Coping Ability Drives Improvements in Mood and Quality of Life for Patients Taking Adjuvant Endocrine Therapy. *Psycho-Oncology*. 2024;33(12):e70049. doi:10.1002/pon.70049

32. Mougalian SS, Epstein LN, Jhaveri AP, et al. Bidirectional Text Messaging to Monitor Endocrine Therapy Adherence and Patient-Reported Outcomes in Breast Cancer. *JCO Clinical Cancer Informatics*. 2017;(1):1-10. doi:10.1200/CCI.17.00015

35. Okuyama H, Takada F, Taira N, Nakamura S. A randomized trial of the impact of symptom monitoring using an electronic patient-reported outcome app on health-related quality of life in postmenopausal breast cancer patients receiving adjuvant endocrine therapy. *Breast Cancer*. 2024;31(5):787-797. doi:10.1007/s12282-024-01592-4

27. Brett J, Boulton M, Watson E. Development of an e-health app to support women prescribed adjuvant endocrine therapy after treatment for breast cancer. *PPA*. 2018;Volume 12:2639-2647. doi:10.2147/PPA.S187692

28. Harbeck N, Kates R, Schinköthe T, et al. Favorable impact of therapy management by an interactive eHealth system on severe adverse events in patients with hormone receptor-positive, HER2-negative locally advanced or metastatic breast cancer treated by palbociclib and endocrine therapy. *Cancer Treatment Reviews*. 2023;121:102631. doi:10.1016/j.ctrv.2023.102631

42. Richardson D, Zhan L, Mahtani R, et al. A prospective observational study of patient-reported functioning and quality of life in advanced and metastatic breast cancer utilizing a novel mobile application. *Breast Cancer Res Treat*. 2021;187(1):113-124. doi:10.1007/s10549-020-06082-7

33. Hershman DL, Unger JM, Hillyer GC, et al. Randomized Trial of Text Messaging to Reduce Early Discontinuation of Adjuvant Aromatase Inhibitor Therapy in Women With Early-Stage Breast Cancer: SWOG S1105. *JCO*. 2020;38(19):2122-2129. doi:10.1200/JCO.19.02699

45. Aurell C, Haidar A, Giglio D. Follow-up Routines Matter for Adherence to Endocrine Therapy in the Adjuvant Setting of Breast Cancer. *Breast Cancer�(Auckl)*. 2024;18:11782234241240171. doi:10.1177/11782234241240171

50. Tan EH, Wong ALA, Tan CC, et al. Improving medication adherence with adjuvant aromatase inhibitor in women with breast cancer: A randomised controlled trial to evaluate the effect of short message service (SMS) reminder. *The Breast*. 2020;53:77-84. doi:10.1016/j.breast.2020.06.012

30. Neuner J, Weil E, Fergestrom N, et al. Feasibility of a pharmacist-led symptom monitoring and management intervention to improve breast cancer endocrine therapy adherence. *Journal of the American Pharmacists Association*. 2022;62(4):1321-1328.e3. doi:10.1016/j.japh.2022.03.001

36. Advani P, Brewster AM, Baum GP, Schover LR. A pilot randomized trial to prevent sexual dysfunction in postmenopausal breast cancer survivors starting adjuvant aromatase inhibitor therapy. *J Cancer Surviv*. 2017;11(4):477-485. doi:10.1007/s11764-017-0606-3

43. Harrell M, Fabbri D, Levy M. Analysis of Adjuvant Endocrine Therapy in Practice From Electronic Health Record Data of Patients With Breast Cancer. *JCO Clinical Cancer Informatics*. 2017;(1):1-8. doi:10.1200/CCI.16.00044

44. Yin Z, Harrell M, Warner JL, Chen Q, Fabbri D, Malin BA. The therapy is making me sick: how online portal communications between breast cancer patients and physicians indicate medication discontinuation. *Journal of the American Medical Informatics Association*. 2018;25(11):1444-1451. doi:10.1093/jamia/ocy118

54. Nathan M, Beatty L, Coborn J, et al. Targeting psychological and menopausal factors linked to non‐adherence to anti‐estrogen therapy in women with early‐stage hormone‐receptor positive breast cancer: A web‐based feasibility study of Finding My Way. *Psycho-Oncology*. 2023;32(9):1461-1465. doi:10.1002/pon.6195

49. Wheeler SB, Spencer J, Drier SW, Fray N, Reeder-Hayes KE. Motivational Interviewing Counseling to Increase Endocrine Therapy Adherence in Diverse Patients. *Cancers*. 2023;15(7):1973. doi:10.3390/cancers15071973

47. Krukowski RA, Hu X, Arshad S, et al. Symptom Monitoring App Use Associated With Medication Adherence Among Woman Survivors of Breast Cancer on Adjuvant Endocrine Therapy. *JCO Clin Cancer Inform*. 2024;(8):e2400179. doi:10.1200/CCI-24-00179
